# Sex-Specific Associations of Trimethylamine-N-Oxide and Zonulin with Signs of Depression in Carbohydrate Malabsorbers and Nonmalabsorbers

**DOI:** 10.1155/2020/7897240

**Published:** 2020-01-06

**Authors:** Sophie Meinitzer, Andreas Baranyi, Sandra Holasek, Wolfgang J. Schnedl, Sieglinde Zelzer, Harald Mangge, Markus Herrmann, Andreas Meinitzer, Dietmar Enko

**Affiliations:** ^1^Clinical Institute of Medical and Chemical Laboratory Diagnostics, Medical University of Graz, 8036 Graz, Austria; ^2^Department of Psychiatry and Psychotherapeutic Medicine, Medical University of Graz, 8036 Graz, Austria; ^3^Department of Immunology and Pathophysiology, Medical University of Graz, 8010 Graz, Austria; ^4^Practice for General Internal Medicine, 8600 Bruck/Mur, Austria; ^5^Institute of Clinical Chemistry and Laboratory Medicine, General Hospital Hochsteiermark, 8700 Leoben, Austria

## Abstract

**Background:**

The microbiome-derived trimethylamine-N-oxide (TMAO) and the intestinal permeability marker zonulin are considered to be linked with depression. Moreover, carbohydrate malabsorption (CMA) was shown to be associated with signs of depression. This study is aimed at investigating possible sex-specific associations between TMAO and zonulin and the presence of depressive signs in individuals with and without CMA.

**Methods:**

Serum concentrations of TMAO and zonulin were determined in 115 and 136 individuals with the presence or absence of CMA. All 251 study participants underwent lactase gene C/T_−13910_ polymorphism genotyping and fructose H_2_/CH_4_ breath testing. Additionally, they filled in the Beck Depression Inventory (BDI-II) questionnaire.

**Results:**

The median TMAO and zonulin serum concentrations were 2.66 (1.93–4.14) *μ*mol/L and 40.83 (34.73–47.48) ng/mL. Serum TMAO levels were positively correlated with depressive symptoms (*P* = 0.011, *ρ* = 0.160). The strongest correlations were observed in 87 females (*P* = 0.010, *ρ* = 0.274) and 49 males (*P* = 0.027, *ρ* = 0.315) without CMA, whereas 115 patients with CMA showed no significant correlations. Zonulin tended to be negatively correlated with the BDI-II score in 49 males without CMA (*P* = 0.062, *ρ* = −0.269).

**Conclusion:**

This study demonstrates a positive correlationship between the serum TMAO concentrations and the severity of depressive symptoms in females and males without CMA. Serum zonulin levels were negatively correlated with signs of depression in males without CMA. These findings suggest a gender-specific relationship between the serum TMAO and zonulin concentrations, depression, and CMA.

## 1. Introduction

In recent years, the gut-brain axis, which consists of interactions between the gut microbiome and the brain, has gained substantial attention. The main principal mechanisms of this complex bidirectional communication comprise modulation of the gut-blood barrier (GBB), alteration in intestinal motility, mucosal immunoregulation, and the production of local neurotransmitters and bacterial metabolites [1].

The gut bacteria-derived metabolite trimethylamine-N-oxide (TMAO) is a small organic molecule, which was recently shown to be associated with neurodegenerative diseases [2] and postoperative cognitive dysfunction [3]. The gut microbiome metabolizes phosphatidyl and L-carnitine containing nutrients (i.e., eggs, red meat, cheese, and seasalt fish) to trimethylamine (TMA), which is absorbed into the bloodstream and then oxidized by the hepatic flavin-containing monooxygenase (FMO) family members FMO-1 and FMO-3 [4, 5]. Therefore, the serum TMAO levels depend on many influencing factors including nutrition, gut microbiome composition, liver function, and the GBB, which regulates the permeability of gut-derived molecules into the blood circulation [6].

The GBB permeability is physiologically regulated by the human protein zonulin, which interacts with a specific intestinal surface epithelial receptor and triggers an intracellular cascade of biochemical events with the subsequent induction of intercellular tight junction disassembly [7, 8]. Alterations of the GBB function are associated with functional gastrointestinal conditions (i.e., irritable bowel syndrome, food malabsorption, infections, genetic predisposition, and stress) [9]. Food malabsorption may be often secondarily the result of chronic inflammatory processes in the gastrointestinal tract with subsequent increased permeability [9, 10].

Carbohydrate malabsorption (CMA) is a frequent and widespread gastrointestinal condition leading to the intestinal accumulation of undigested lactose and fructose, which reach the large intestine, where colonic bacteria generate degradation products such as short-chain fatty acids, carbon dioxide (CO_2_), hydrogen (H_2_), and methane (CH_4_) [11, 12]. It is hypothesized that the nonabsorbed carbohydrates, especially fructose, might lead to alterations in the intestinal wall with subsequent local inflammation and increased intestinal permeability [13]. Moreover, in previously published studies CMA was reported to be associated with signs of depression [14, 15].

Recently, it was shown that depression is also linked with concentration changes of the gut bacteria-derived metabolite TMAO [16, 17] and the GBB biomarker zonulin [18]. Nevertheless, study designs investigating a possible gender-specific link of TMAO and zonulin with signs of depression in populations grouped by CMA and non-CMA are not available yet.

The aim of this study was to investigate possible sex-specific associations between the intestinal biomarkers TMAO and zonulin and the Beck Depression Inventory (BDI-II) in individuals subgrouped by the presence or absence of CMA.

## 2. Materials and Methods

### 2.1. Study Design

A total of 251 consecutive ambulatory patients, who were admitted to the outpatient clinic for CMA testing, were included in this study. All study participants underwent *LCT* genotyping (C/T_−13910_ polymorphism) and fructose H_2_/CH_4_ breath testing to identify individuals with lactose and fructose malabsorption. Blood sampling was performed after an overnight fasting state in the morning between 08:00 and 10:00 a.m. evaluating the serum concentrations of TMAO and zonulin. Additionally, all study participants filled in the Beck Depression Inventory (BDI-II) questionnaire [19].

The inclusion criteria for this study were consecutive individuals referred to our outpatient clinic with suspected CMA, a minimum age of 18 years, and an obligatory overnight fasting and nonsmoking period. Individuals with an antibiotic-based therapy or colonoscopy at least 4 weeks before the H_2_/CH_4_ breath test and patients with an acute or chronic inflammatory bowel disease were excluded from the study.

All study participants provided their written informed consent. Approval was obtained from the local Ethical Committee of the Johannes Kepler University Linz (Linz, Austria). The study was carried out in accordance to the current version of the Declaration of Helsinki.

### 2.2. Carbohydrate MA Testing

To detect individuals with primary adult-type lactose malabsorption, *LCT* genotyping (C/T_−13910_ polymorphism) was performed [12]. VACUETTE® K3EDTA tubes (2 mL) (Greiner Bio-One International GmbH, Kremsmünster, Austria) were drawn from patients for genomic DNA extraction on a MagNA Pure Compact Instrument (Roche Diagnostics, Rotkreuz, Switzerland). Real-time PCR with specific fluorescent labelled hybridization probes, followed by a melting curve analysis (*LCT* T-13910C ToolSet™ (Roche Diagnostics)), was established on a LightCycler® 2.0 Instrument (Roche Diagnostics).

A fructose breath test protocol was established to detect patients with fructose malabsorption. Gas chromatography was employed to measure the H_2_ and CH_4_ concentration using a QuinTron Model DP Plus MicroLyzer™ (QuinTron, Milwaukee, WI, USA). After determining baseline breath H_2_/CH_4_ concentrations, fructose was given in a dose of 25 g dissolved in 200 mL of water. The end-expiratory breath H_2_/CH_4_ concentrations were measured at 15, 30, 45, 60, 75, 90, and 120 minutes after fructose ingestion. Patients were classified with fructose malabsorption, if a H_2_ and/or CH_4_ increase >20 ppm above baseline concentrations was observed [11, 12, 20].

### 2.3. Determination of Intestinal Biomarkers TMAO and Zonulin

Fasting blood samples were drawn into 4 mL VACUETTE® Z Serum Clot Activator tubes (Greiner Bio-One International GmbH, Kremsmünster, Austria). Serum samples were centrifuged at 1800 x g for 10 minutes and immediately analyzed. TMAO was measured using a stable-isotope-dilution assay and high-performance liquid chromatography (HPLC) with electrospray ionization tandem mass spectrometry on a SCIEX QTRAP 4500 triple quadrupole instrument (Applied Biosystems, Framingham, MA, USA) equipped with an Agilent 1260 Infinity HPLC system (Agilent Technologies, Santa Clara, CA, USA). The intra- and interday coefficients of variation (CVs) ranged between 2.2–5.5% and 7.6–9.9%, respectively.

The serum zonulin concentrations were measured with the IDK® enzyme linked immunoassay (ELISA) (Immunodiagnostic AG, Bensheim, Germany) according to the manufacturer's recommendations. The intraday CVs were <8% and the interday CVs were <12%, respectively.

### 2.4. BDI-II Score

For the assessment of the presence and severity of depressive symptoms, we used the BDI-II score. This questionnaire consists of 21 items (scale: 0–3), and the total values range between 0 and 63 (21). According to the literature, individuals with a BDI-II score > 13 were classified with the presence of depressive symptoms [21, 22].

### 2.5. Statistical Analysis

Descriptive statistics were computed for the categorical and metric variables. The Kolmogorov-Smirnov test was performed to calculate data distribution. As continuous variables were not normally distributed, they were expressed as medians with interquartile ranges (Q1–Q3). Categorical variables were expressed as percentages. The exact Mann-Whitney *U*-test was performed for subgroup comparisons of nonnormal distributed metric variables. Spearman's rho (*ρ*) was calculated to assess possible positive or negative correlations between the intestinal biomarkers TMAO and zonulin and the BDI-II score. A *P* value of <0.05 was considered statistically significant. For all statistical analyses, the SPSS 25.0 statistical software (SPSS Inc., Chicago, IL, USA) was used.

## 3. Results

### 3.1. General Characteristics of the Study Population

The clinical and laboratory characteristics of the study population are shown in Table 1. The average age was 40.6 ± 14.5 years. Of all 251 study participants, 160 (63.7%) were female and 91 (36.3%) were male. In total, 115 (73 females and 42 males) (45.8%) patients were identified with CMA. Of these, 50 (19.9%) patients showed lactose malabsorption, 50 (19.9%) showed fructose malabsorption, and 15 (6%) were diagnosed with lactose and fructose malabsorption.

Sixty-five (56.5%) out of 115 individuals with CMA were C/C_−13910_ homozygotes, the *LCT* genotype indicative of primary-adult lactose malabsorption, and 32 (27.8%) and 18 (15.7%) individuals could be identified as C/T_−13910_ hetero- and T/T_−13910_ homozygotes. The genotype frequencies for 136 non-CMA individuals were 86 (63.2%) for the C/T_−13910_ and 50 (36.8%) for the T/T_−13910_ genotype.

The median BDI-II score (interquartile ranges) was 10 (3–20). Overall, 155 (95 females and 60 males) (61.8%) and 96 (65 females and 31 males) (38.2%) individuals were found with a BDI-II score ≤ 13 and >13, respectively.

### 3.2. TMAO and Zonulin Measurements and BDI-II Score

**Figure 1:**
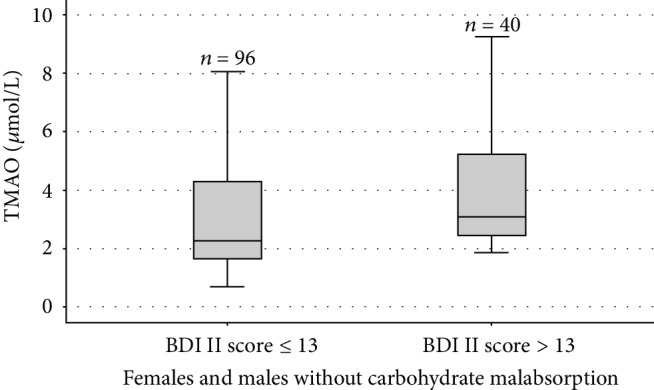
Box-and-whisker plot of trimethylamine-N-oxide (TMAO) comparisons between 96 and 40 individuals without CMA with a Beck Depression Inventory (BDI-II) score ≤ 13 and >13. The central boxes represent the 25–75^th^ percentile range. The lines inside the boxes show the median value for each group (*P* = 0.002).

**Figure 2:**
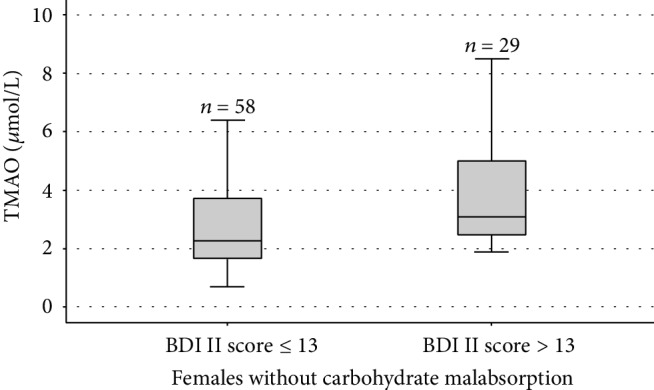
Box-and-whisker plot of trimethylamine-N-oxide (TMAO) comparisons between 58 and 29 females without CMA with a Beck Depression Inventory (BDI-II) score ≤ 13 and >13. The central boxes represent the 25–75^th^ percentile range. The lines inside the boxes show the median value for each group (*P* = 0.003).

**Figure 3:**
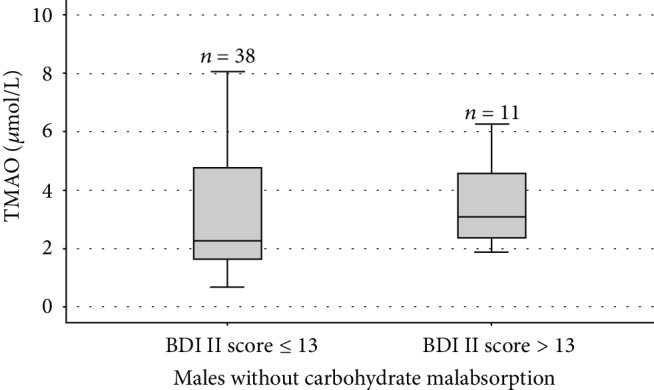
Box-and-whisker plot of trimethylamine-N-oxide (TMAO) comparisons between 38 and 11 males without CMA with a Beck Depression Inventory (BDI-II) score ≤ 13 and >13. The central boxes represent the 25–75^th^ percentile range. The lines inside the boxes show the median value for each group (*P* = 0.259).

The median TMAO and zonulin serum concentrations were 2.66 (1.93–4.14) *μ*mol/L and 40.83 (34.73–47.48) ng/mL. The correlations of TMAO and zonulin with the BDI-II score are illustrated in Table 2. In the study population (*n* = 251), a positive correlation was observed between the TMAO and the BDI-II score (*P* = 0.011, *ρ* = 0.160). The serum TMAO concentrations were positively correlated with the BDI-II score in individuals with non-CMA (*n* = 136; *P* = 0.001, *ρ* = 0.277), both females (*n* = 87; *P* = 0.010, *ρ* = 0.274) and males (*n* = 49; *P* = 0.027, *ρ* = 0.315). The TMAO serum concentration comparison between 96 and 40 non-CMA individuals with a BDI-II score ≤ 13 and >13 is shown in Figure 1. Gender-specific TMAO level comparisons in females and males between BDI-II score ≤ 13 and >13 are illustrated in Figures 2 and 3.

Zonulin tended to be negatively correlated with the BDI-II score in males without CMA (*n* = 49; *P* = 0.062, *ρ* = −0.269).

### 3.3. Correlations of BMI with Zonulin and TMAO

There was a positive correlation between BMI and zonulin (all patients: *P* < 0.001, *ρ* = 0.266; females: *P* = 0.003, *ρ* = 0.238; males: *P* = 0.004, *ρ* = 0.302). No statistically relevant correlation was observed between BMI and TMAO.

## 4. Discussion

In the current work, we investigated 251 ambulatory individuals, who were primarily referred for CMA testing to our outpatient clinic for possible gender-specific associations between TMAO and zonulin concentrations and the BDI-II score. To the best of our knowledge, this is the first study addressing this intestinal biomarker profile in individuals with and without CMA. We found a significantly positive correlationship for TMAO serum concentrations and the severity of depressive symptoms (*P* = 0.011, *ρ* = 0.160). The strongest association was observed in males without CMA (*P* = 0.027, *ρ* = 0.315) followed by females without CMA (*P* = 0.010, *ρ* = 0.274), whereas individuals with CMA showed no significant correlations.

In comparison, a previously published study by Liu et al., in which 16 depressed patients and 16 healthy subjects were recruited, reported significantly higher (*P* < 0.05) TMAO blood levels in the depressed group compared to the healthy control group [23]. The studied patient groups were too small for the assessment of possible gender-specific differences. Another previous study indicated that depressed individuals have an altered gut microbiome metabolism, including concentration changes of metabolites, such as dimethylamine, dimethylglycine, and TMAO [16].

These findings emphasize the potential influence of the gut microbiome in the development of depression. However, the exact pathophysiological mechanism of the gut-brain axis cannot be fully explained yet. The gut microbiota-derived TMAO needs to pass the GBB, which is a complex intestinal multilayer system regulating the absorption of water and nutrients [6]. Since TMAO was shown to be present in human cerebrospinal fluid [24], it is hypothesized that TMAO could have the potential to cross the BBB [25]. These findings are indicative that TMAO could play a direct role in the central nervous system [24].

Here, correlations between TMAO and the BDI-II score differed between CMA and non-CMA. CMA is known as a gastrointestinal condition associated with changes of the gut microbiome composition and leaky intestinal barrier function [26–28]. This could be a potential explanation for the various correlationships between TMAO blood levels and depressive symptoms found here. Present data also indicate distinctive gender-specific differences between the relationship of TMAO and depressive symptoms with strongest associations found in males without CMA.

In males without CMA, serum zonulin levels tended to be negatively correlated with depressive symptoms (*P* = 0.062, *ρ* = −0.269). Recently, Ohlsson et al. showed that depressive symptoms correlated negatively at trend with zonulin blood concentrations (*P* = 0.07, *r* = −0.21) [29]. However, differences in the study designs and participants must be mentioned: Ohlsson et al. compared 54 individuals with recent suicide attempts with 13 subjects with major depressive disorders and no history of a suicide attempt and 17 healthy controls. For the assessment of depressive symptoms, the authors used the Montgomery-Åsberg Depression Rate Scale (MADRS) [29]. In contrast, we assessed depressive symptoms with the BDI-II score in 115 and 136 individuals with and without CMA and additionally considered gender-specific variations.

Our data suggest that serum zonulin levels in males may represent a different pattern of gut integrity compared to females. A recently published study showed that zonulin has the potential to enhance the permeability of both the GBB and the BBB [30]. Zonulin causes a dysregulation of the endothelial tight junctions of these barriers [10]. As a consequence of the altered permeability, the development of depressive symptoms may be triggered by microbiota-derived degradation products (i.e., neurotransmitters, TMAO, and toxic bacterial metabolites) [31].

Nevertheless, it must me mentioned that the measurement of serum zonulin as a biomarker of leaky gut with currently commercially available ELISA kits is limited because of the lack of protein specificity of monoclonal capture antibodies [32, 33].

Two limitations of this study may be described. Firstly, this study is cross-sectional in nature, representing serum TMAO and zonulin assessment at a measuring single point, only. Secondly, secondary acquired and reversible forms of lactose malabsorption were not identified because the lactose H_2_/CH_4_ breath test was not performed in this study.

## 5. Conclusions

In conclusion, females and males without CMA showed a positive correlationship between TMAO serum concentrations and the severity of depressive symptoms. Serum zonulin concentrations were negatively correlated in males without CMA. These findings are indicative of a sex-specific link between the intestinal biomarkers TMAO and zonulin and depressive symptoms in individuals without malabsorption. However, the authors suggest performing further prospective longitudinal studies to fully elucidate the relationship between sex-specific differences of the TMAO and zonulin pattern, CMA and depressive disorders.

## Figures and Tables

**Table 1:** Baseline characteristics of the study population (*n* = 251).

Characteristic	Baseline
Age (yr)	40.6 ± 14.5
Sex	
Female	160 (63.7%)
Male	91 (36.3%)
Carbohydrate malabsorption	
Lactose malabsorber	65 (25.9%)
Fructose malabsorber	65 (25.9%)
Nonmalabsorber	136 (54.2%)
Laboratory parameters	
TMAO (*μ*mol/L)	2.66 (1.93–4.14)
Zonulin (ng/mL)	40.83 (34.73–47.48)
Depressive symptoms	
BDI-II score	10 (3–20)

TMAO: trimethylamine-N-oxide; BDI-II: Beck Depression Inventory. Data are presented as means ± standard deviation, or medians (Q1–Q3), or percentage.

**Table 2:** Sex-specific correlations of TMAO and zonulin with the BDI-II score in individuals with and without CMA.

	Study population (*n* = 251)	CMA (*n* = 115)	CMA (females = 73)	CMA (males = 42)	Non-CMA (*n* = 136)	Non-CMA (females = 87)	Non-CMA (males = 49)
	TMAO	TMAO	TMAO	TMAO	TMAO	TMAO	TMAO
BDI-II score	0.160^a^	0.040	0.009	0.128	0.277	0.274	0.315
	0.011^b,^^∗^	0.671	0.941	0.417	0.001^∗^	0.010^∗^	0.027^∗^

	Zonulin	Zonulin	Zonulin	Zonulin	Zonulin	Zonulin	Zonulin
BDI-II score	0.012	0.035	0.029	0.043	0.037	0.191	- 0.269
	0.854	0.712	0.810	0.785	0.666	0.077	0.062

BDI-II: Beck Depression Inventory; CMA: carbohydrate malabsorption; TMAO: trimethylamine-N-oxide. Spearman's correlation analysis was calculated. ^a^Spearman's rho (*ρ*); ^b^*P* value. ^∗^A *P* value < 0.05 was considered statistically significant.

## Data Availability

The data used to support the findings of this study are available from the corresponding author upon request.
